
JOURNAL INFORMATION
==============================
NLM Title Abbreviation: ScientificWorldJournal
Journal ID: ScientificWorldJournal
NLM Journal ID: 101131163
Journal ID: TSWJ

The Scientific World Journal

ISSN: 2356-6140
EISSN: 1537-744X
Publisher: Wiley

ARTICLE INFORMATION
==============================
PMCID: PMC5936513
PMID: 29861680
DOI: 10.1100/tsw.2005.62
Article ID: 368390
Article version: 1
Subjects: Editorial

Microscopy-Aims and Scope

Tzaphlidou Margaret
University of Ioannina, Medical School, Department of Medical Physics, Ioannina, Greece
Publication date: 2005
Electronic publication date: 2005 Jun 3
Volume: 5
First page: 470
Last page: 470
Copyright: Copyright © 2005 Margaret Tzaphlidou.
Copyright year: 2005
License: This is an open access article distributed under the Creative Commons Attribution License, which permits unrestricted use, distribution, and reproduction in any medium, provided the original work is properly cited.
License URL: https://creativecommons.org/licenses/by/3.0/

==============================
Editorial
*TheScientificWorldJOURNAL* (2005) 5, 470
ISSN 1537-744X; DOI 10.1100/tsw.2005.62

### Microscopy - Aims and Scope

Margaret Tzaphlidou
*University of Ioannina, Medical School, Department of Medical Physics, Ioannina, Greece*

E-mail: mtzaphli@cc.uoi.gr

*Published June 3, 2005*

---

The aim of the **Microscopy** domain is to serve as a forum for all research that involves the application, practice, and theory of microscopy and microanalysis as well as basic research linked to clinical medicine. The domain will also give strong emphasis to computer image processing and any other image analytical methods.

**Microscopy** will embrace original research work in Biophysics and general in Physical Sciences, Biology and Medicine as well as review articles. The Editors will invite particular articles on various aspects of microscopy and image processing, although all review article submissions are welcome.

From time-to-time special issues from conferences will be published and special themed issues edited by experts in the field are also especially welcome.

---

**This article should be referenced as follows:**

Tzaphlidou, Margaret. (2005) Microscopy-Aims and Scope. *TheScientificWorldJOURNAL* **5**, 470.

---

*Corresponding author.
©2005 with author.
